# Integrated multi-omics reveals the molecular mechanisms underlying efficient phosphorus use under phosphate deficiency in elephant grass (*Pennisetum purpureum*)

**DOI:** 10.3389/fpls.2022.1069191

**Published:** 2022-12-23

**Authors:** Jiajia Luo, Zeping Cai, Rui Huang, Yuanhang Wu, Chun Liu, Chunqiong Huang, Pandao Liu, Guodao Liu, Rongshu Dong

**Affiliations:** ^1^ Tropical Crops Genetic Resources Institute, Chinese Academy of Tropical Agricultural Sciences, Haikou, China; ^2^ College of Forestry and College of Tropical Crops, Hainan University, Haikou, China

**Keywords:** phosphate deficiency, *pennisetum purpureum*, transcriptome, metabolome, molecular mechanism

## Abstract

Phosphorus (P) is an essential macronutrient element for plant growth, and deficiency of inorganic phosphate (Pi) limits plant growth and yield. Elephant grass (*Pennisetum purpureum*) is an important fodder crop cultivated widely in tropical and subtropical areas throughout the world. However, the mechanisms underlying efficient P use in elephant grass under Pi deficiency remain poorly understood. In this study, the physiological and molecular responses of elephant grass leaves and roots to Pi deficiency were investigated. The results showed that dry weight, total P concentration, and P content decreased in Pi-deprived plants, but that acid phosphatase activity and P utilization efficiency (PUE) were higher than in Pi-sufficient plants. Regarding Pi starvation-responsive (PSR) genes, transcriptomics showed that 59 unigenes involved in Pi acquisition and transport (especially 18 *purple acid phosphatase* and 27 *phosphate transporter 1* unigenes) and 51 *phospholipase* unigenes involved in phospholipids degradation or Pi-free lipids biosynthesis, as well as 47 core unigenes involved in the synthesis of phenylpropanoids and flavonoids, were significantly up-regulated by Pi deprivation in leaves or roots. Furthermore, 43 unigenes related to Pi-independent- or inorganic pyrophosphate (PPi)-dependent bypass reactions were markedly up-regulated in Pi-deficient leaves, especially five *UDP-glucose pyrophosphorylase* and 15 *phosphoenolpyruvate carboxylase* unigenes. Consistent with PSR unigene expression changes, metabolomics revealed that Pi deficiency significantly increased metabolites of Pi-free lipids, phenylpropanoids, and flavonoids in leaves and roots, but decreased phospholipid metabolites. This study reveals the mechanisms underlying the responses to Pi starvation in elephant grass leaves and roots, which provides candidate unigenes involved in efficient P use and theoretical references for the development of P-efficient elephant grass varieties.

## 1 Introduction

Phosphorus (P) is an essential nutrient required for plant growth, and is a major component of cellular macromolecules and a crucial element of metabolism (Lambers, 2021). Inorganic phosphate (Pi) is the only form of P that can be absorbed directly by plants. Unfortunately, low Pi availability is a significant constraint on agricultural production, because Pi is readily immobilized and precipitated as insoluble Pi complexes in soil (López-Arredondo et al., 2014). López-Arredondo et al. (2014) have reported that Pi deficiency is a feature of ~70% of global cultivated lands. Thus, excessive amounts of Pi fertilizers are applied to optimize crop yields, but only 30% can be taken up by crops, and the rest is lost through runoff, leaching, and water erosion into ground and surface waters or fixed in soils (Cong et al., 2020). Veneklaas et al. (2012) have reported that agricultural P runoff is a major source of environmental pollution. Furthermore, Pi fertilizer is mainly obtained from non-renewable and finite rock P, and global P resources could be depleted within 50–100 years (Chowdhury et al., 2017). Thus, P fertilizer overuse, P resource scarcity, and P pollution threaten food security and ecological development of agriculture. These P issues are closely linked with the ineffective use of P (Dissanayaka et al., 2021). In this regards, in-depth understanding of P use mechanisms will provide information to develop more P-efficient crops.

Plants’ P use efficiency has been divided into P acquisition efficiency (PAE) and P utilization deficiency (PUE) (Ding et al., 2020). Plant PAE is the capacity for Pi uptake from the soil by roots. In response to Pi deprivation, plants have evolved multiple strategies to enhance P acquisition, including regulating the activity of Pi transporters (PHTs) to increase Pi uptake, enhancing root exploration for Pi, and improving Pi scavenging (e.g., through phosphatase and ribonuclease) (Cong et al., 2020; Ding et al., 2020). Purple acid phosphatases (PAPs) are involved in the remobilization not only of external organic P (extracellular type PAPs), but also of intracellular organic P (intracellular type PAPs) (Farhadi et al., 2020). Previous studies have reported that PAPs play important roles in response to Pi starvation in various plant species, such as *Oryza sativa*, *Zea mays*, *Glycine max*, and *Cicer arietnum* (Zhang et al., 2011; Li et al., 2012; González-Muñoz et al., 2015; Bhadouria et al., 2017). Pi transporter 1 (*PHT1*) family genes are believed to be associated with Pi uptake and translocation (Roch et al., 2019). In addition, flavonoids can directly mobilize Pi from rhizosphere insoluble inorganic P (e.g., Fe-Pi and Al-Pi) (Ding et al., 2020). Multidrug and toxic compound extrusion (MATE) proteins perform various functions in plants, and MATE subfamily II (MATE II) members reportedly use flavonoids as substrates, and are involved in flavonoids transport (Wang et al., 2016).

Plant PUE is the capacity to produce total biomass or yield by per unit P, also represented by the proficiency of Pi recycling in plants (Dissanayaka et al., 2018). The PUE of Pi-deprived plants can be increased by enhancing the remobilization and recycling of internal Pi. Firstly, vacuolar phosphate influx transporter (VPE) functions as a Pi-mobilizing enzyme to transport vacuolar Pi into the cytosol to enhance Pi recycling in Pi-deprived plants (Dissanayaka et al., 2021). Secondly, nucleic acids represent ~50% of organic P in a plant cell, especially ribonucleic acid (RNA), and ribonucleases (RNSs) degrade RNA to release Pi, to increase Pi recycling in response to Pi starvation in plants (Dissanayaka et al., 2021). Furthermore, membrane lipid remodeling is an important response to enhance Pi recycling under low-Pi stress, and this process involves two main steps; phospholipid degradation to liberate Pi and phospholipid replacement by Pi-free lipids (major galactolipids and sulfolipids) (Dissanayaka et al., 2018; Ding et al., 2020). Moreover, regulation bypass enzymes in glycolytic metabolism can reduce Pi use and alleviate low-Pi stress; for example, inorganic pyrophosphate (PPi)-dependent and Pi-independent glycolytic enzymes replace Pi- and adenylate-dependent enzymes (Cong et al., 2020; Dissanayaka et al., 2021).

Elephant grass (*Pennisetum purpureum*) is a perennial C_4_ plant of the Poaceae family, and is primarily cultivated as an important fodder crop in tropical and subtropical areas of Brazil, America, and Africa (Yan et al., 2020). In addition, elephant grass is the most widely cultivated tropical forage in south China, and forage grass shortage caused by the novel coronavirus epidemic has aggravated the cultivation of elephant grass in recent years. Although elephant grass is characterized by high biomass production, with plants reaching a height of 2–6 m, a yield of up to 45 t/ha green matter, and allowing three or four cuttings per year, this crop requires a large amount of P fertilizer (Ferreira et al., 2022). Therefore, development of high P efficiency elephant grass cultivars could reduce the application of P fertilizer, resulting in more environmentally friendly and sustainable agricultural production (Zhang et al., 2022). Previous studies related to elephant grass have mainly focused on the development of bioenergy (such as bioethanol and biodiesel), phytoremediation of soils polluted by heavy metals (such as cobalt and chromium), and tolerance to abiotic stresses (such as high temperature, drought, and low fertility) (Anderson et al., 2008; Lotfy and Mostafa, 2014; Yan et al., 2020). However, the molecular mechanisms underlying efficient P use under Pi deficiency in elephant grass have not been reported.

Herein, we investigated changes in genes and metabolites induced by Pi deficiency by combining transcriptome and metabolome analyses, to explore efficient use P strategies and mine critical genes in Pi-deficient elephant grass. The findings provide candidate genes and lay a theoretical foundation for developing more P-efficient elephant grass cultivars.

## 2 Material and methods

### 2.1 Plant treatment and total P measurement

Elephant grass was provided by the Chinese Academy of Tropical Agricultural Sciences (CATAS), Hainan, China. In a previous study, Huang et al. (2021) found that dry weight, total P content, acid phosphatase (APase) activity, and expression levels of three *PAP* genes of elephant grass were significantly altered by Pi deficiency for 10 days. Therefore, we conducted the present study based on Huang et al. (2021) to identify the genes and metabolites associated with P use in response to low-Pi stress in elephant grass. Briefly, elephant grass stems were sprouted for 7 days, and uniform seedlings were transferred to Magnavaca’s solution, as described by Famoso et al. (2010). The hydroponic culture solution was supplemented with 600 μM KH_2_PO_4_ and adjusted to pH 5.80, and plants were cultivated in a greenhouse with a temperature range of 26−35°C under a natural day–night cycle. After 10 days of preculture, seedlings were treated with 0 μM KH_2_PO_4_ (low-Pi treatment, LP) or 600 μM KH_2_PO_4_ (high-Pi treatment, HP). For LP treatment, 600 μM KCl was instead added to supplement potassium. After 10 days of Pi treatment, elephant grass shoots and roots were harvested for dry weight and total P measurement, with each treatment including six biological replicates, and each biological replicate including one seedling. For dry weight analysis, samples were oven dried at 75°C. For total P analysis, ~0.05 g of each shoot or root sample powder was burned to ash at a temperature of 600°C using a muffle furnace. The total P concentration was determined by phosphorus–molybdate blue color reaction, and the optical density at 700 nm (OD_700_) value of reaction products was measured by spectrophotometry (Bio-Rad, CA, USA), as described by Murphy and Riley (1962). Finally, P content and P utilization efficiency were calculated depending on the dry weight and total P concentration of plant samples, as described previously (Chen et al., 2021). After 10 days of Pi treatment, mixed leaves (including upper, middle, and lower leaves) and whole roots were harvested for APase activity, transcriptome, and metabolome analyses, with each treatment including three biological replicates (four seedlings from each treatment were combined to form one biological replicate). The APase activity of Pi-deficient samples was measured using ρ-nitrophenylphosphate as substrate, as described previously (Luo et al., 2020).

### 2.2 RNA extraction and full-length transcriptomic analysis

For transcriptomic analysis, total RNA was extracted from three biological replicate samples of leaves and roots under LP or HP treatments (total 12 samples) using RNeasy Mini kits (Tiangen Biotech, Beijing, China). The concentration and quality of total RNA were assessed by a Nanodrop 2000c Spectrophotometer (ThermoFisher Scientific, Waltham, MA, USA) and an Agilent 2100 Bioanalyzer (Agilent Technologies, CA, USA), respectively. Furthermore, equal amounts of RNA were taken from 12 samples and mixed them together for full-length transcriptomic sequencing, and a cDNA library was constructed using a SMARTer PCR cDNA Synthesis Kit (Clontech, Terra Bella, CA, USA). DNA sequencing was performed on a PacBio Sequel II platform. Circular consensus sequences (CCSs) were identified using SMRTlink software (version 10.2; http://ccs.how/) and redundant CCSs were filtered by CD-HIT software (version 4.8.1;Fu et al., 2012). Non-redundant isoforms were generated after clustering and polishing of CCSs.

### 2.3 Comparative transcriptome analysis

From the 12 samples, cDNA libraries were generated using an NEBNext Ultra RNA Library Prep Kit (NEB, PA, USA). Libraries were sequenced on an Illumina NovaSeq platform (Illumina, CA, USA), and 150-bp paired-end (PE150) reads were produced with an insert size ~350 bp. All raw reads were subjected to filtering of low-quality and adaptor reads using the filter module of SOAPnuke (version 2.16). Clean reads were further mapped back to the full-length isoforms database using bowtie2 (version 2.2.5). Genes expression levels in each sample were calculated using RSEM (version 1.3.3), and normalized to the fragments per kilobase million mapped reads (FPKM). For annotation, all expressed isoform sequences were aligned by BLAST search (E-value <1E^–5^) against Nr and Swiss-Prot (protein sequence databases), Nt (nucleotide sequence database), KEGG (Kyoto Encyclopedia of Genes and Genome), KOG (Clusters of orthologous groups for eukaryotic complete genomes), Pfam (Protein Family), and GO (Gene Ontology) databases. To identify differentially expressed genes (DEGs), the DESeq2 (version 1.32.0) package in R software (version 4.0.2) was employed to calculate gene expression differences between LP and HP treatments, and genes with |log_2_(LP/HP)| > 1 and adjusted *P*-value (*P*_adj_) < 0.05 were defined as DEGs. Thus, DEGs with log_2_(LP/HP) > 1 or log_2_(LP/HP) < –1 were defined as up-regulated and down-regulated, respectively. Subsequently, KEGG and GO enrichment analysis were executed using the R platform, as previously described (Tai et al., 2018), and KEGG pathways or GO terms with *P*_adj_ < 0.05 were defined as significantly enriched.

### 2.4 Identification of Pi starvation-responsive (PSR) genes and phylogenetic analysis

Using the protein sequences encoded by PSR genes in the model plants *Arabidopsis thaliana* (TAIR, www.arabidopsis.org) and *O. sativa* (https://data.jgi.doe.gov/) as references, elephant grass proteins were subjected to homology-based searching to predict the presence of PSR genes; genes with a BLASTP (version 2.9.0) alignment E-value < 1E^–10^ (indicating coverage of >35% in *A. thaliana* and *O. sativa*) were identified as candidate genes (Liu et al., 2022). In this study, we searched for the following genes in elephant grass:

* PSR genes involved in Pi acquisition and transport, such as genes of the *PAP*, *RNS*, *PHT1*, and *VPE* families;
* genes involved in the membrane phospholipid remodeling metabolic pathway, such as the genes coding for phospholipase A (PLA), phospholipase D (PLD), phosphatidate phosphatase (PAH), and glycerophosphodiester phosphodiesterase (GDPD);
* genes involved in the alternative pathway of cytosolic glycolysis, such as those encoding sucrose synthase/UDP-glucose pyrophosphorylase (UGPase), PPi-dependent 6-phosphofructokinase (PPi-PFK), and phosphoenolpyruvate carboxylase (PEPC);
* genes implicated in the flavonoid metabolic pathway, such as those encoding phenylalanine ammonia-lyase (PAL), 4-coumarate-CoA ligase (4CL), trans-cinnamate 4-monooxygenase (C4H), chalcone synthase (CHS), and members of the MATE II family of proteins.
* PSR genes of *A. thaliana* and *O. sativa* are listed in **Supplementary Table S18**.

Phylogenetic analysis of *PAP* and *PHT1* family genes was performed using the amino acid sequences of *A. thaliana* and *O. sativa*, and the identified differentially expressed isoforms in elephant grass. Multiple sequence alignment was performed by ClustalX software, and a neighbor-joining phylogenetic tree was constructed with 1,000 bootstrap replicates using MEGA7 software.

### 2.5 Lipidomics analysis

To investigate changes in the abundance of lipids, a targeted lipid metabolomics study was performed at BGI Co. Ltd (Shenzhen, China). Lipid metabolites were extracted from all 12 samples, and levels were measured using ultra performance liquid chromatography–tandem mass spectrometry (UPLC-MS/MS) technology as previously described (McLoughlin et al., 2018). Briefly, 25 mg of each sample was extracted in dichloromethane/methanol (3:1 v/v, –20°C precooling), and the supernatant was lyophilized and resuspended before analysis. Subsequently, lipids in 5-μl extracts of each sample were separated by a CSH C18 column (Waters, MA, USA). Under positive ion mode, the mobile phase consisted of solvent A (0.1% v/v formic acid + 10 mM ammonium formate + 60% v/v acetonitrile aqueous) and solvent B (0.1% v/v formic acid + 10 mM ammonium formate + 10% v/v acetonitrile aqueous + 90% v/v isopropanol). Under negative ion mode, the mobile phases of solvent A and solvent B without 0.1% (v/v) formic acid were the same as in positive ion mode. The flow rate and column temperature were set as 0.35 ml/min and 55°C, respectively. The *m*/*z* range of MS scans was 200−2,000, the flow rates of sheath gas and auxiliary gas were 40 L/min and 10 L/min, and the temperatures of capillary and aux gas heaters were 320°C and 350°C, respectively. Additionally, qualitative and quantitative analyses of lipid metabolites were performed using LipidSearch software (version 4.1) as described by Chauhan et al. (2019). The metaX package in R software (version 4.0.2) was employed to preprocess the original data exported by LipidSearch, including the deletion of lipid molecules missing from >80% of experimental samples, adding missing values using the K-nearest neighbor (KNN) algorithm, and normalization of data by the probabilistic quotient normalization (PQN) method. Finally, differentially accumulated lipids (DALs) were identified based on fold change of LP/HP >2 or <0.5 and *P*_adj_ *<*0.05, where *P*_adj_ represented the *P*-value of *t*-test after false discovery rate (FDR) correction.

### 2.6 Detection of phenylpropanoid and flavonoid metabolites

In leaves and roots of elephant grass under Pi deprivation, targeted determination of phenylpropanoids and flavonoids was performed by UPLC-MS/MS technology at Shanghai Applied Protein Technology Co. Ltd (Shanghai, China). Briefly, 100 mg of each sample was extracted using methanol, acetonitrile, and water (2:2:1 v/v/v) by ultrasonic-assisted extraction, and the supernatant was retained. Extracts (2 μl) of each sample were loaded onto the UPLC-MS/MS system, and liquid chromatography analysis was performed using an ACQUITY UPLC system (Waters) equipped with a BEH C18 column (Waters). The mobile phase composition and gradient elution conditions were consistent with those reported by Norazhar et al. (2021). Additionally, MS/MS analysis was performed using a QTrap 5500 tandem MS instrument (AB SCIEX, MA, USA). The concentrations of metabolites were quantified by the standard curve method, and standard metabolites were brought from Sigma-Aldrich and Steraloids. Differentially accumulated metabolites (DAMs) were defined by a *P*-value <0.05, and we used a *t*-test to assess the significance of differences between two comparison pairs, including LPL vs. HLP and LPR vs. HLR.

### 2.7 Quantitative real-time PCR (qRT-PCR) analysis

Total RNA from elephant grass leaf and root tissues was extracted using TRNZol reagent (Tiangen) in accordance with the manufacture’s protocol. First-strand cDNA synthesis from total RNA was performed using a HiScript II 1st Strand cDNA Synthesis Kit plus gDNA eraser (Vazyme, Nanjing, China). Subsequently, qRT-PCR was carried out using ChamQ Universal SYBR qPCR Master Mix (Vazyme) on a QuantStudio 6 Flex qRT-PCR system (Applied Biosystems, Waltham, MA, USA) following the manufacturer’s instructions. Twenty DEGs were selected for analysis by qRT-PCR, and the *Actin1* gene (gene ID *PpACT1*, isoform26256, NCBI accession number MT784734) served as a reference gene to normalize gene expressions, as described by Huang et al. (2021). Primers used to amplify genes for qRT-PCR analysis are listed in **Supplementary Table S23**. Relative expression values were calculated by the 2^-ΔΔCT^ method (An et al., 2022).

### 2.8 Statistical analysis

Data analysis was conducted in Excel 2016 (Microsoft Corporation, Redmond, WA, USA). The significance of difference was tested by Student’s *t*-test using SPSS software (version 19.0; IBM, USA). In addition, the R program (version 4.2.0) was employed to visualize the results of principal component analysis (PCA) and to generate volcano diagrams and heatmaps. The co-expression networks were constructed using Cytoscape (version 3.6.1).

### 2.9 Data availability

The sequencing data of full-length transcriptome and comparative transcriptome are deposited in the Sequence Read Archive (SRA) of the NCBI database, under Bioproject accession number PRJNA854591.

## 3 Results

### 3.1 Changes in dry weight, total phosphorus, and acid phosphatase activity in response to Pi deficiency

**Figure 1:**
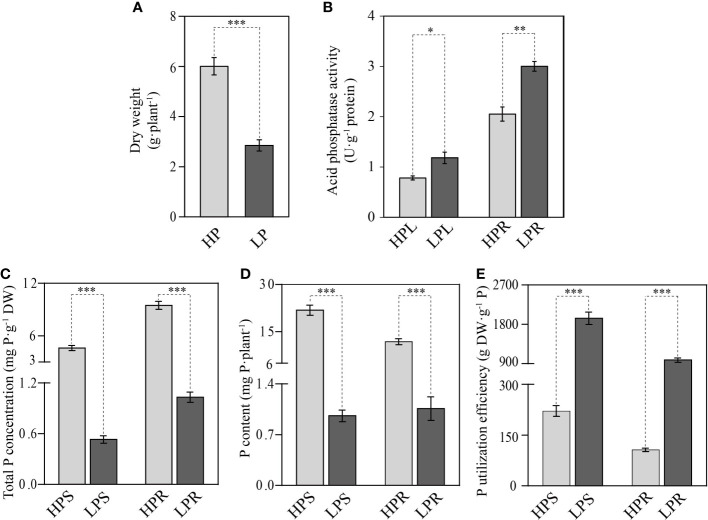
Effects of Pi starvation on elephant grass (*Pennisetum purpureum*). **(A)** Dry weight of total plant. **(B)** Acid phosphatase activity of leaves and roots. **(C–E)** Total P concentration **(C)**, P content **(D)**, and P utilization efficiency **(E)** of shoots and roots. The uniform seedlings were treated with (HP) or without (LP) 600 μmol/L KH_2_PO_4_ for 10 days. HPL, HPS, and HPR indicate, respectively, leaves, shoots, and roots under the HP condition LPL, LPS, and LPR indicate, respectively, leaves, shoots, and roots under the LP condition,. The data are displayed as means and standard error (SE). Significant differences between different treatments are marked by asterisks: *P <0.05, **P <0.01, ***P <0.001.

In this work, in accordance with the study of Huang et al. (2021), elephant grass was treated with 0 μM KH_2_PO [low Pi supply (LP), i.e., Pi deficient] or 600 μM KH_2_PO_4_ [high Pi supply (HP), i.e., Pi sufficient] for 10 days. The results showed that Pi deficiency had a significant impact on elephant grass growth. The whole-plant dry weight, total P concentration, and P content in Pi-deficient leaves and roots were significantly decreased, by 52.5%–95.6%, relative to Pi-sufficient plants (**Figures 1A, C, D**). Conversely, the APase activities of leaves and roots were increased by 33.8% and 31.6%, respectively, under Pi deficiency stress (**Figure 1B**). In addition, PUE was increased by 7.8- and 8.2-fold in Pi-deficient leaves and roots, respectively, compared with Pi-sufficient plants (**Figure 1E**). These results indicate that Pi deficiency resulted in reduced biomass accumulation, decreased total P storage, and enhanced PUE in elephant grass.

### 3.2 Transcriptome analysis under Pi deprivation conditions

Full-length and comparative transcriptome sequencing of elephant grass leaves and roots was carried out. In the full-length transcriptome data, we identified 93,953 transcripts, which were grouped into 88,715 unigenes (**Supplementary Table S1**). Subsequently, RNA sequencing (RNA-seq) was performed to analyze the global expression profile of unigenes under Pi starvation. The full-length transcriptome was used as a reference. RNA-Seq results are displayed in **Supplementary Table S2**. A total of 53,595 unigenes expressed in more than one sample were identified; the annotation and expression levels of these unigenes are given in **Supplementary Tables S3**,**S4**, respectively.

**Figure 2:**
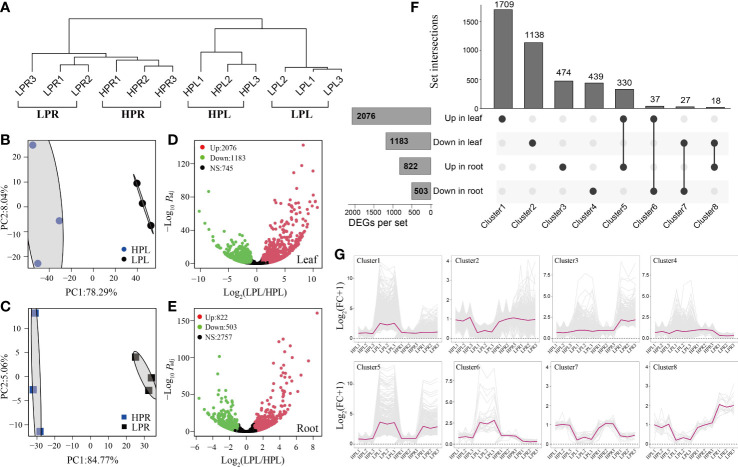
Overview of differentially expressed unigenes (DEGs) in the transcriptome of elephant grass. **(A)** Hierarchical clustering of all samples based on expressed genes in the transcriptome. **(B, C)** Principal component analysis of all expressed unigenes in leaves **(B)** and roots **(C)**. **(D, E)** Volcano diagrams of distribution of unigenes in Pi-deficient leaves **(D)** and roots **(E)**. **(F)** Specific and overlapping DEGs in Pi-deficient leaves and roots. **(G)** The differential expression patterns of DEGs in Clusters 1–8. The fold change of each unigene was normalized to the mean generated with all HP samples. LPL and LPR represent, respectively, leaves and roots treated with 0 μmol/L KH_2_PO_4_. HPL and HPR represent, respectively, leaves and roots treated with 600 μmol/L KH_2_PO_4_, the same as below.

Hierarchical clustering and PCA showed that Pi starvation profoundly influenced the transcriptomes of elephant grass leaves and roots (**Figures 2A-C**). We identified 2,076 up-regulated unigenes and 1,183 down-regulated unigenes in elephant grass leaves and 822 up-regulated unigenes and 503 down-regulated unigenes in elephant grass roots (**Figures 2D-F**; **Supplementary Table S5**). A total of 4,172 DEGs expressed in at least one tissue were detected, including 3,760 specific DEGs and 412 overlapping DEGs (**Figure 2F**). The specific and overlapping DEGs were divided into clusters 1−4 and clusters 5−8, respectively (**Figure 2F**). Cluster 1 (up-regulated only in leaves) had the most specific DEGs and Cluster 5 (up-regulated in leaves and roots) had the most overlapping DEGs (**Figure 2F**). The differential expression patterns of DEGs in Clusters 1−8 are shown in **Figure 2G**.

**Figure 3:**
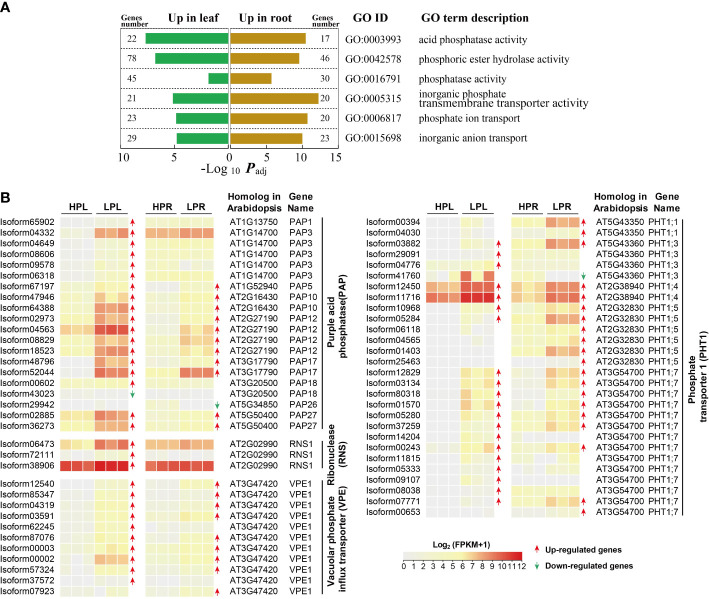
Expression changes of unigenes associated with Pi acquisition and transport in elephant grass upon Pi deprivation. **(A)** The significantly enriched KEGG pathways and GO terms associated with differentially expressed phosphate acquisition and transport-related unigenes. **(B)** Differentially expressed unigenes encoding purple acid phosphatase (PAP), ribonuclease (RNS), vacuolar phosphate influx transporter (VPE), and phosphate transporter 1 (PHT1) in leaves and roots of elephant grass under Pi starvation.

**Figure 4:**
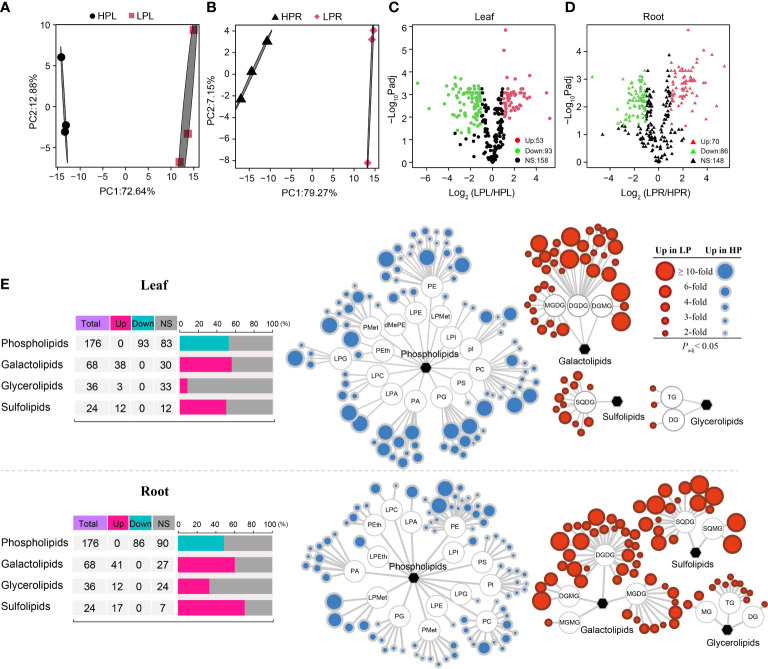
Analysis of lipid metabolome in elephant grass leaves and roots upon Pi starvation. **(A, B)** Principal component analysis of all lipid metabolites in elephant grass leaves **(A)** and roots **(B)**. **(C, D)** Volcano diagrams of lipids in Pi-deficient leaves **(C)** and roots **(D)** of elephant grass. **(E)** Statistic classification and profile of differentially expressed lipids in Pi-deficient leaves and roots. DGDG, digalactosyldiacylglycerol; MGDG, monogalactosyldiacylglycerol; DGMG, digalactosylmonoacylglycerol; MGMG, monogalactosylmonoacylglycerol; SQDG, sulfoquinovosyldiacylglycerol; SQMG, sulfoquinovosylmonoacylglycerol; TG, triglyceride; DG, diglyceride; MG, monoglyceride; PG, phosphatidylglycerol; PI, phosphatidylinositol; PA, phosphatidic acid; PEth, phosphatidylethanol; PS, phosphatidylserine; LPMet, lyso-phosphatidylmethanol; LPG, lyso-phosphatidylglycerol; LPC, lyso-phosphatidylcholine; LPE, lyso-phosphatidylethanolamine; LPI, lyso-phosphatidylinositol; LPA, lyso-phosphatidic acid; LPEth, lyso-phosphatidylethanol; dMePE, dimethylphosphatidyethanolamine; PE, phosphatidylethanolamine; PC, phosphatidylcholine; PMet, phosphatidylmethanol.

We performed KEGG and GO enrichment analysis of all DEGs (genes up-regulated and down-regulated in leaves and roots of elephant grass subjected to Pi starvation; **Supplementary Tables S6**−**S16**). In the KEGG analysis, metabolic pathways (ko01100), glycerophospholipid metabolism (ko00564), and glycolysis/gluconeogenesis (ko00010) were significantly enriched (**Figure S1**; **Supplementary Tables S6**, **S7**, **S9**, and **S10**). Consistent with the KEGG results, the significantly enriched terms in GO analysis were also associated with Pi acquisition and transport, glycerophospholipid metabolism, and glycolysis metabolism (**Figures 3A**, **4A**, and **6A**; **Supplementary Tables S12**, **S13**, **S15**, and **S16**).

### 3.3 Phosphate acquisition- and transport-related genes in response to Pi deficiency

GO enrichment analysis showed that all up-regulated unigenes in Pi-deprived leaves and roots were associated with Pi acquisition and transport (**Figure 3A**; **Supplementary Tables S13** and **S16**). Subsequently, we identified *PAPs*, *RNSs*, *VPEs* and *PHT1s* in elephant grass, based genes of *A. thaliana* and *O. sativa* (**Supplementary Table S18**). A total of 48 *PAP* genes were identified, including 18 up-regulated genes and one down-regulated gene in leaves, and 11 up-regulated genes and one down-regulated gene in roots (**Figure 3B**; **Supplementary Tables S19** and **S20**). There were 11 co-up-regulated *PAP* genes in leaves and roots under Pi deficiency, including one *PAP5* unigene, two *PAP10* unigenes, four *PAP12* unigenes, two *PAP17* unigenes, and two *PAP27* unigenes (**Figure 3B**). Furthermore, we identified 12 *RNS* unigenes and three *RNS* unigenes up-regulated only in Pi-deficient leaves (**Figure 3B**; **Supplementary Tables S19** and **S20**). Additionally, 18 *VPE1* unigenes were identified, including 10 *VPE1* and nine *VPE1* unigenes up-regulated in leaves and roots, respectively, and eight *VPE1* unigenes were co-up-regulated in leaves and roots under Pi starvation (**Figure 3B**; **Supplementary Table S19**, **S20**). Finally, 55 *PHT1s* were identified, including 20 up-regulated in leaves, 20 up-regulated in roots, and one down-regulated in roots (**Figure 3B**; **Supplementary Tables S19** and **S20**). Thirteen co-up-regulated *PHT1* unigenes in leaves and roots under Pi deficiency were identified: eight *PHT1;7*, two *PHT1;5*, two *PHT1;4*, and one *PHT1;3* unigenes (**Figure 3B**).

Furthermore, 18 differentially expressed *PpPAP* genes (two shorter isoform67197 and isoform48796 genes were excluded), 29 *AtPAP* genes, and 26 *OsPAP* genes were used to construct a protein phylogenetic tree. The tree showed that six *PpPAP* genes, three *PpPAP* genes, seven *PpPAP* genes, and two *PpPAP* genes belonged to the IIIb, IIb, Ia-2, and Ib-2 subfamilies, respectively (**Figure S3A**). The protein phylogenetic tree of 28 differentially expressed *PpPHT1* genes, nine *AtPHT1* genes, and 13 *OsPHT1* genes showed that two PpPHT1 unigenes, one PpPHT1, three PpPHT1s, four PpPHT1s, three *PpPHT1* genes, and 15 *PpPHT1* genes were closely related to *OsPHT1;11*, *OsPHT1;6*, *OsPHT1;8*, *OsPHT1;3*, *OsPHT1;1*, and *OsPHT1;2*, respectively (**Figure S3B**).

### 3.4 Lipidomics analysis upon Pi deficiency

In order to evaluate changes in lipid metabolites in response to Pi starvation, we performed lipid metabolomics analysis on leaves and roots of elephant grass. A total of 304 metabolites were identified in elephant grass under Pi starvation (**Supplementary Table S21**). PCA revealed significant variation in metabolites between Pi-sufficient and Pi-deficient leaves (**Figure 4A**), as well as between Pi-sufficient and Pi-deficient roots (**Figure 4B**). Subsequently, we identified 146 DALs (53 up-regulated and 93 down-regulated) in Pi-deficient leaves and 156 DALs (70 up-regulated and 86 down-regulated) DALs in Pi-deficient roots (i.e., accumulated lipids that differed from those found in Pi-sufficient tissues) (**Figures 4C, D**). Furthermore, all DALs were classified into Pi-containing and Pi-free groups (**Supplementary Table S21**). We observed that all 93 down-regulated lipids in Pi-deficient leaves and 86 down-regulated lipids in Pi-deficient roots belonged to the Pi-containing group (phospholipids), mainly phosphatidylethanolamine (PE), phosphatidylcholine (PC), and phosphatidylglycerol (PG) (**Figure 4E**). A total of 53 and 70 lipids increased in abundance in Pi-deprived leaves and roots, respectively. These belonged mainly to the Pi-free group, and included the galactolipids digalactosyldiacylglycerol (DGDG), monogalactosyldiacylglycerol (MGDG),and digalactosylmonoacylglycerol (DGMG), the sulfolipids sulfoquinovosylmonoacylglycerol (SQDG), and sulfoquinovosylmonoacylglycerol (SQMG, and the glycerolipids triglyceride (TG), diglyceride (DG), and monoglyceride (MG), as shown in **Figure 4E**.

### 3.5 Changes in lipids in response to Pi deficiency

**Figure 5:**
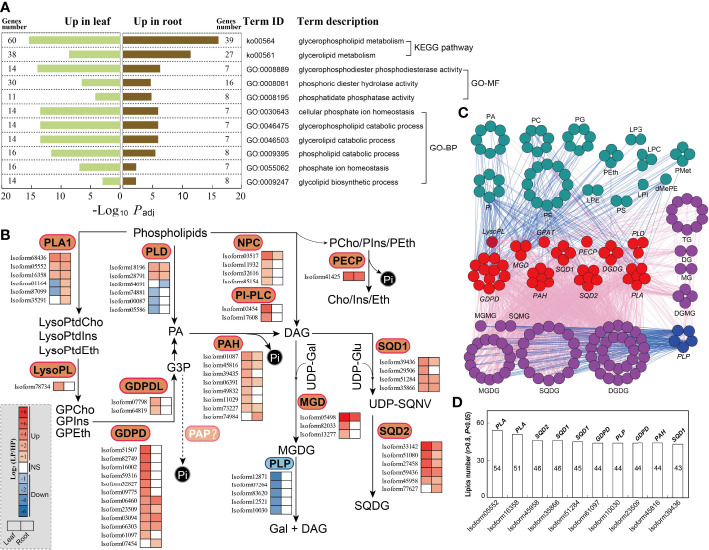
Phospholipid metabolism responding to Pi deficiency in elephant grass leaves and roots. **(A)** The significantly enriched KEGG pathways and GO categories associated with DEGs of phospholipid metabolites. **(B)** DEGs associated with phospholipid metabolism are shown; color intensity corresponds to expression levels standardized to log_2_(LP/HP) scales. **(C)** Association analysis of phospholipid metabolism DEGs and differentially expressed lipids (|*r*| >0.8, *P <*0.05). Dark-green, purple, red, and blue circles indicate decreased and increased lipids and up-regulated and down-regulated unigenes, respectively. Blue and pink lines indicate negative (*r* <–0.8, *P <*0.05) and positive (*r >*0.8, *P <*0.05) correlation, respectively. **(D)** The top 10 unigenes most correlated with lipids (data based on Figure 5C). PLA1, phospholipase A1; LysoPL, lysophospholipase; GDPD, glycerophosphodiester phosphodiesterase; GDPDL, glycerophosphodiester phosphodiesterase-like; PLD, phospholipase D; PAH, phosphatidate phosphatase; NPC, non-specific phospholipase C; PI-PLC, phosphoinositide phospholipase C; PECP, phosphoethanolamine/phosphocholine phosphatase; MGD, monogalactosyl diacylglycerol synthase; SQD1, sulfoquinovosyldiacylglycerol 1; SQD2, sulfoquinovosyldiacylglycerol 2.

In KEGG and GO enrichment analyses, we identified several dominant pathways and terms associated with phospholipid metabolism (**Figure 5A**; **Supplementary Tables S6**, **S7**, **S9**, **S10**, **S12**, **S13**, **S15**, and **S16**), including glycerophospholipid metabolism (ko00564), glycerolipid metabolism (ko00561), and glycerophospholipid catabolic processes (GO:0046503). Subsequently, we identified genes involved in phospholipid metabolism in elephant grass, based on homologs in *A. thaliana* (**Supplementary Tables S18** and **S19**). The results showed that some unigenes encoding phospholipid-hydrolyzing enzymes were up-regulated upon Pi starvation, including the *PLA1*, *PLD*, and *NPC* unigenes (**Figure 5B**; **Supplementary Table S20**). Moreover, we found some up-regulated unigenes associated with diacylglycerol (DAG) generation, including the *lysophospholipase* (*LysoPL*) and *GDPD* unigenes (**Figure 5B**; **Supplementary Table S20**). Furthermore, Pi starvation enhanced the expression levels of phosphomonoesterase genes, the protein products of which function in the hydrolysis of phosphate monoester bonds to produce Pi (**Figure 5B**; **Supplementary Table S20**), including the *PAH*, *phosphoethanolamine*/*phosphocholine phosphatase* (*PECP*), and *PAP* unigenes, which may be associated with the hydrolysis of glycerol 3-phosphate (G3P). Finally, we observed some up-regulated unigenes related to Pi-free lipid accumulation, such as the *sulfoquinovosyldiacylglycerol 1* (*SQD1*) and *sulfoquinovosyldiacylglycerol 2* (*SQD2*) unigenes, which play roles in sulfoquinovosyldiacylglycerol (SQDG) synthesis, and *monogalactosyl diacylglycerol synthase* (*MGD*) unigenes that are responsible for the generation of monogalactosyl diacylglycerol (MGDG). At the same time, five *patatin-like protein* (*PLP*) unigenes were down-regulated in leaves, and *PLPs* mainly function in the degradation of MGDG (**Figure 5B**; **Supplementary Table S20**). In addition, correlation analysis revealed that Pi-containing lipids (such as PC, PG, and PE) and Pi-free lipids (mainly MGDG, SQDG, and DGDG) were, respectively, significantly negatively and positively associated with unigenes related to phospholipid degradation (such as *PLA*, *PLD*, and *GDPD*) and Pi-free lipid synthesis (such as *MGD*, *SQD1*, and *SQD2*), and positively and negatively associated with *PLP* unigenes (**Figure 5C**). Among the top 10 unigenes *PLA* (isoform05552) was significantly correlated with the most lipids, and *SQD1* (isoform39436) was significantly correlated with the fewest lipids. Thus, the top 10 DEGs might play important roles in the lipid remodeling pathway under low-Pi stress in elephant grass (**Figure 5D**).

### 3.6 Glycolysis metabolism in response to Pi starvation

**Figure 6:**
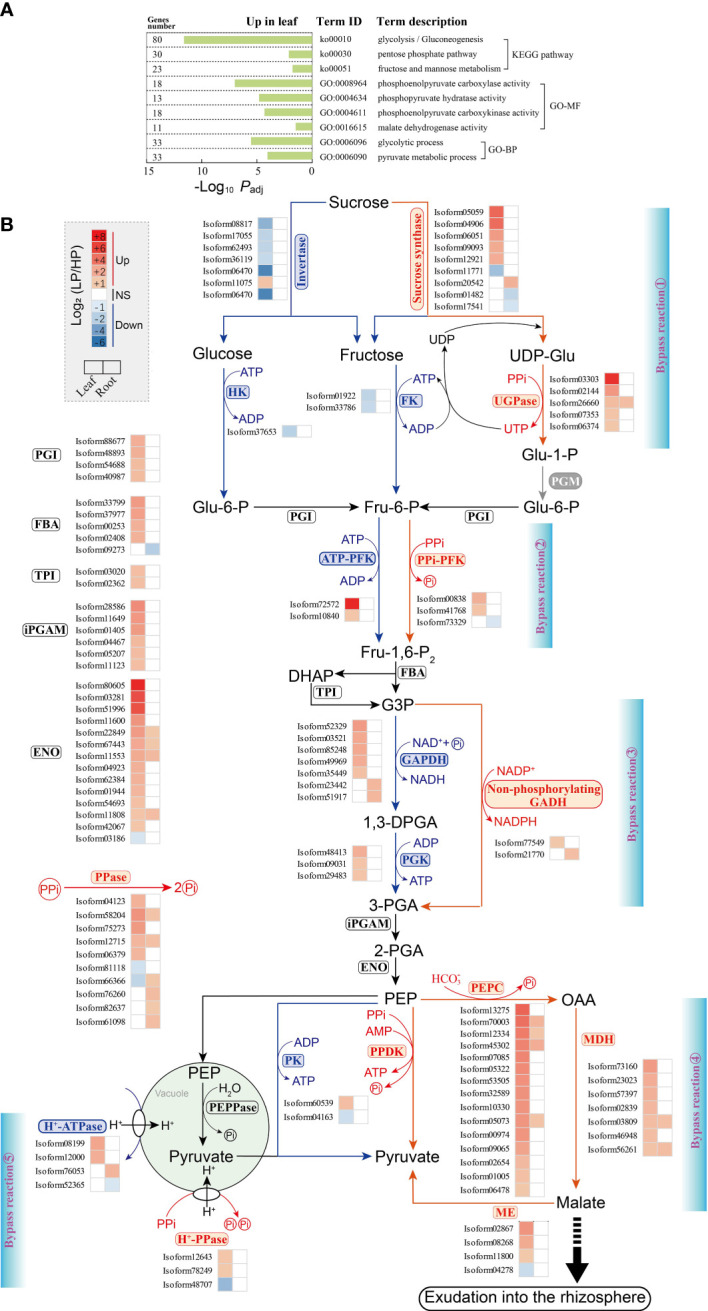
Changes in unigenes involved in glycolysis in elephant grass leaves and roots upon Pi deprivation. **(A)** The significantly enriched KEGG pathways and GO terms associated with DEGs of glycolysis metabolism. **(B)** DEGs associated with glycolysis metabolism are shown; color intensity corresponds to expression levels calculated using log_2_(LP/HP). The five bypass reactions performed by Pi-independent or PPi-dependent enzymes, are marked in red. UGPase, UDP-glucose pyrophosphorylase; FK, fructokinase; HK, hexokinase; PPi-PFK, PPi-dependent 6-phosphofructokinase; ATP-PFK, ATP-dependent 6-phosphofructokinase; FBA, fructose-bisphosphate aldolase; TPI, triosephosphate isomerase; GAPDH, NAD-dependent glyceraldehyde-3-phosphate dehydrogenase; PGK, phosphoglycerate kinase; non-phosphorylating GADH, NADP-dependent glyceraldehyde-3-phosphate dehydrogenase; iPGAM, 2,3-bisphosphoglycerate-independent phosphoglycerate mutase; ENO, enolase; PK, pyruvate kinase; PPDK, pyruvate orthophosphate dikinase; PEPC, phosphoenolpyruvate carboxylase; MDH, malate dehydrogenase; ME, malic enzyme; PEPPase, phosphoenolpyruvate phosphatase; H^+^-ATPase, H^+^-adenosine triphosphatase; H^+^-PPase, H^+^-pyrophosphatase; PPase, inorganic pyrophosphatase.

In KEGG and GO enrichment analyses, we found that DEGs were grouped into several dominant pathways and terms associated with glycolysis metabolism in elephant grass leaves, but not in roots, such as glycolysis/gluconeogenesis (ko00010), pentose phosphate (ko00030), and glycolytic process (GO:0006096) pathways (**Figure 6A**; **Figures S1** and **S2**; **Supplementary Tables S6**, **S7**, **S12**, and **S13**). Subsequently, Pi starvation-related genes involved in glycolysis metabolism were identified by homology comparison (**Supplementary Tables S18** and **S19**).

In this study, five bypass reactions were generated from ATP- and Pi-dependent glycolytic enzymes bypassed by PPi-dependent enzymes in Pi-deprived leaves (**Figure 6B**). The five bypass reactions were further evidenced by the up-regulation of bypass unigenes. We observed that five *sucrose synthase* and five *UGPase* unigenes were up-regulated in Pi-deprived leaves (**Figure 6B**; **Supplementary Table S20**). Conversely, six *invertase*, one *hexokinase* (*HK*), and two *fructokinase* (*FK*) unigenes were down-regulated in Pi-deprived leaves (**Figure 6B**; **Supplementary Table S20**).

In addition, the second, third, and fifth bypass reactions revealed, respectively, that expression levels of two *PPi-PFK*, one *NADP-dependent glyceraldehyde-3-phosphate dehydrogenase* (*non-phosphorylating GADH*), and two *H^+^-pyrophosphatase* (*H^+^-PPase*) unigenes in leaves were up-regulated during Pi starvation (**Figure 6B**; **Supplementary Table S20**). Four ATP-dependent unigenes (two *ATP-dependent 6-phosphofructokinase* and two *H^+^*-*ATPase* unigenes) in the second and fifth bypass reactions, as well as Pi-dependent unigenes (five *NAD-dependent glyceraldehyde-4-phosphate dehydrogenase* and three *phosphoglycerate kinase* unigenes) in the third bypass reaction, were found to be up-regulated in Pi-deficient leaves (**Figure 6B**; **Supplementary Table S20**).

Furthermore, as shown in the fourth bypass reaction, 25 bypass unigenes were significantly up-regulated in leaves under Pi starvation, including 15 *PEPC*, seven *malate dehydrogenase* (*MDH*), and three *malic enzyme* (*ME*) unigenes. In contrast to these bypass unigenes, one Pi-dependent unigene (*pyruvate kinase*, *PK*) was down-regulated in Pi-deficient leaves. However, the expression levels of PPi-dependent unigenes (*pyruvate orthophosphate dikinase*, *PPDK*) did not respond to Pi starvation (**Figure 6B**; **Supplementary Table S20**). Accordingly, five *PPase* unigenes were observed to be up-regulated in Pi-deficient leaves (**Figure 6B**; **Supplementary Table S20**).

### 3.7 Phenylpropanoid and flavonoid metabolism in response to Pi starvation

**Figure 7:**
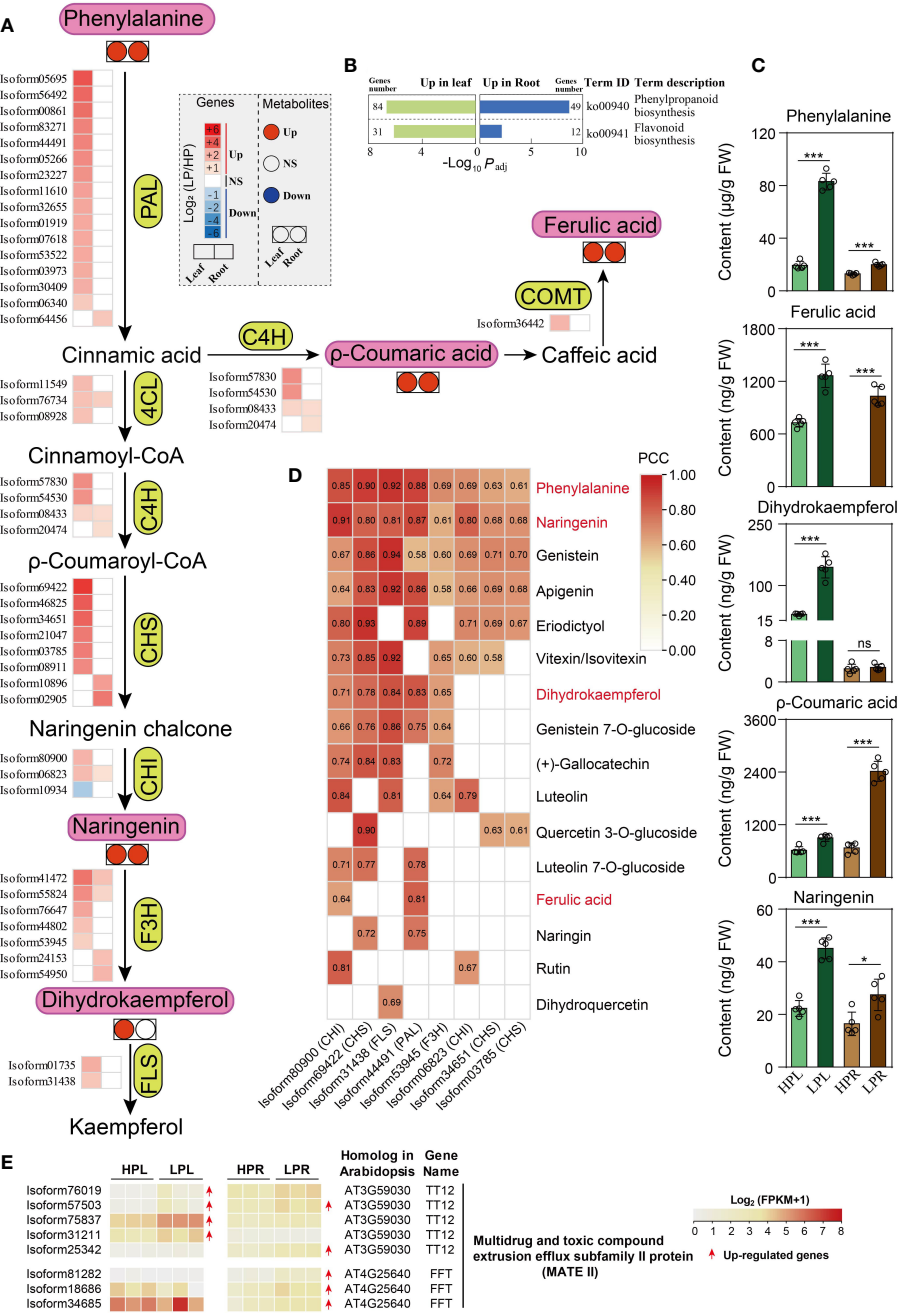
Phenylpropanoids and flavonoids metabolism in response to Pi deficiency in elephant grass leaves and roots. **(A, C)** Changes in the expression levels of critical unigenes **(A)** and the contents of five metabolites **(C)** in leaves and roots. **(B)** The significantly enriched KEGG pathways associated with DEGs of phenylpropanoid and flavonoid metabolism. **(D)** Significantly positive correlation between unigenes and metabolites (*P <*0.05). **(E)** DEGs encoding multidrug and toxic compound extrusion efflux subfamily II (MATE II) proteins in leaves and roots of elephant grass under Pi starvation. PAL, phenylalanine ammonia-lyase; C4H, trans-cinnamate 4-monooxygenase; COMT, flavone 3′-O-methyltransferase; 4CL, 4-coumarate-CoA ligase 3; CHS, chalcone synthase; CHI, chalcone-flavanone isomerase; F3H, naringenin,2-oxoglutarate 3-dioxygenase; FLS, flavonol synthase/flavanone 3-hydroxylase. Asterisks in **(C)** indicate significant differences using the Student’s t-test (*: 0.01 < *P* < 0.05, ***: *P* < 0.001), ns indicate non-significant differences (*P* > 0.05).

According to KEGG enrichment analysis, phenylpropanoid biosynthesis (ko00940) and flavonoid biosynthesis (ko00941) were the most important pathways in elephant grass leaves and roots (**Figure 7B**; **Figure S1A**; **Supplementary Tables S6**, **S7**, **S9**, and **S10**). Subsequently, we identified eight critical gene families and analyzed their expression levels in response to Pi deficiency (**Supplementary Tables S19** and **S20**). Under low-Pi stress, some members of the above eight gene families were up-regulated in Pi-deficient leaves and roots, including *PAL*, *C4H*, and *CHS* unigenes, compared with Pi-sufficient tissues (**Figure 7A**). The up-regulated unigenes were consistent with the accumulation of subsequent phenylpropanoid and flavonoid metabolites (**Figure 7C**; **Supplementary Table S22**).

In total, we identified 19 DAMs in Pi-deficient leaves, including 17 increased (three phenylpropanoids, one flavanone, one flavonol, two isoflavones, one flavanone, five flavones, and four flavonols) and two decreased (one isoflavone and one flavone) metabolites, compared with Pi-sufficient leaves (**Figure 7C**; **Figure S5**; **Supplementary Table S22**). We also identified nine DAMs in Pi-deficient roots, eight increased (three phenylpropanoids, one flavanone, one isoflavone, two flavones, and one flavonol) and one decreased flavone, compared with Pi-sufficient roots (**Figure 7C**; **Figure S5**; **Supplementary Table S22**).

Correlation analysis between the identified eight gene families and DAMs showed that eight unigenes were significantly positively associated with numerous increased flavonoids, suggesting they might play critical roles in regulating flavonoids (**Figure 7D**). Finally, 37 *MATE* II subfamily genes were identified (**Supplementary Table S19**), of which four and five *MATE* unigenes were up-regulated in leaves and roots under Pi starvation, respectively (**Figure 7E**; **Supplementary Table S20**). Only one *MATE* unigene (isoform57503) was co-up-regulated in both leaves and roots upon Pi deficiency (**Figure 7E**; **Supplementary Table S20**).

### 3.8 Validation of comparative transcriptome data by qRT-PCR

To evaluate the reliability of comparative transcriptome results, the relative expression levels of 20 DEGs were verified by qRT-PCR analysis. The results showed that the log_2_FC(qRT-PCR) data and log2FC(RNA-seq) data from both leaf and root tissues displayed a significant positive correlation (*P <*0.001), with Pearson correlation coefficients (PCCs) of 0.845 and 0.883 in leaf and root tissues, respectively (**Figure S4A**). The relative expression levels of the 20 DEGs are shown in **Figure S4B**. In leaf tissues, the expression patterns of 19 DEGs detected by qRT-PCR and RNA-seq were consistent, with isoform36273 (*PpPAP*) the only exception (**Figure S4B**). In root tissues, the expression patterns of 19 unigenes were the same in both qRT-PCR and RNA-seq data, with isoform10030 (*PpPLP*) the only exception (**Figure S4B**). The apparent inconsistent expression patterns of the above two unigenes might be due to low basic expression levels. Overall, the results confirmed that the comparative transcriptome (RNA-seq) data were reliable.

## 4 Discussion

In this study, the growth and P content of elephant grass declined in Pi-deficient leaves and roots, but the APase activities and PUE increased. The results allowed us to explore the mechanisms of efficient P use under Pi deficiency in elephant grass, which revealed associations with facilitating PAE and PUE, including four major aspects: Pi acquisition and transport; Pi recycling from membrane lipid remodeling; ATP saving and Pi recycling from bypassing the glycolytic pathway; and putative Pi mobilization and cell protection from phenylpropanoid and flavonoid metabolites.

### 4.1 Enhancing phosphate acquisition and transport

PAPs are a unique class of APases, the most well-studied gene family contributing to Pi acquisition (Tran et al., 2010). Previous studies reported that 10 out of 26 *OsPAP* genes in *O. sativa*, 11 out of 33 *ZmPAP* genes in *Z. mays*, 23 out of 35 *GmPAP* genes in *G. max*, 20 out of 25 *SlPAP* genes in *Solanum lycopersicum*, and 21 out of 23 *SgPAP* genes in *Stylosanthes guianensis* were up-regulated upon low-Pi stress (Zhang et al., 2011; Li et al., 2012; González-Muñoz et al., 2015; Srivastava et al., 2020; Chen et al., 2021). Consistently, we found that 18 and 11 out of 48 *PAP* unigenes were up-regulated in leaves and roots, respectively, under Pi deficiency in elephant grass (**Figure 3B**). The expression levels of 11 *PAP* unigenes were co-up-regulated in leaves and roots, including two *PAP10*, four *PAP12*, and two *PAP17* unigenes, homologs of *OsPAP10a*, *OsPAP10c*, and *OsPAP3c* in *O. sativa* (**Figure 3B**; **Figure S3A**; **Supplementary Table S20**). Previous studies have demonstrated that AtPAP10 is a primarily extracellular APase, while AtPAP12 is an intracellular and extracellular APase, and these two PAPs function in the utilization and mobilization of intracellular or extracellular organic P in *A. thaliana* (Wang et al., 2014). *OsPAP10a* and *OsPAP10c* of *O. sativa*, *GmPAP7a* and *GmPAP7b* of *G. max*, and *PvPAP3* of *Phaseolus vulgaris* are involved in extracellular ATP utilization in Pi-deficient roots (Liang et al., 2010; Zhu et al., 2020; Bhadouria and Giri, 2022). Moreover, *OsPAP3* is induced by Pi starvation in flag leaves, and might be involved in seed P loading (Jeong et al., 2017). *AtPAP17* is markedly induced by Pi starvation to maintain Pi homeostasis by remobilizing Pi from phosphoesters in *A. thaliana* senescing leaves (Farhadi et al., 2020). Additionally, nucleic acids are a major source of organic P, particularly RNA, and Pi recycling/scavenging from exogenous or endogenous nucleic acids in response to low-Pi stress has been well documented (Dissanayaka et al., 2021). Pi is released from RNA by RNSs, and previous studies have reported that vacuole ribosome RNAs (rRNAs) were degraded by up-regulated *RNSs* to adapt low-Pi stress in plants (Yang et al., 2017; Dissanayaka et al., 2021). Consistent with this, we also identified three *RNS* unigenes that were up-regulated in elephant grass leaves by Pi deficiency (**Figure 3B**), and these might play roles in Pi recycling/scavenging from RNAs.

PHT1 family proteins are Pi influx transporters that mediate Pi uptake and translocation (López-Arredondo et al., 2014). Previous studies documented that most *PHT1* genes were induced in several plant roots, including *StPHT1;7* of potato (*Solanum tuberosum*) as well as *OsPHT1;1*, *OsPHT1;2*, and *OsPHT1;3* of *O. sativa* (Roch et al., 2019; Zhang et al., 2021). In the present study, we identified 20 *PHT1* unigenes that were up-regulated in elephant grass roots by Pi deprivation, including *PHT1;7*, *PHT1;5*, *PHT1;4*, *PHT1;3*, and *PHT1;1* (**Figure 3B**). Therefore, these low-Pi up-regulated *PHT1* genes might help facilitate Pi uptake in elephant grass roots.

Besides playing a role in Pi uptake, PHT1 proteins may be involved in Pi redistribution (Dai et al., 2022). For example, *AtPHT1;5* of *Arabidopsis*, *OsPHT1;3* of *O. sativa*, and *HvPHT1;6* of barley (*Hordeum vulgare*) are mainly expressed in senescing or old leaves to mediate Pi redistribution (Preuss et al., 2010; Chang et al., 2019; Roch et al., 2019). *OsPHT1;2* of *O. sativa* plays a role in Pi translocation in plants (Ai et al., 2009). In the present study, we identified 20 up-regulated *PHT1* genes in Pi-deficient leaves of elephant grass, including 13 *PHT1;7*, two *PHT1;5*, two *PHT1;4*, and three *PHT1;3* unigenes (**Figure 3B**). We predicted that these up-regulated *PHT1* unigenes in leaves might play a significant role in Pi remobilization. In addition, we identified 13 co-up-regulated *PHT1* unigenes in leaves and roots of elephant grass upon Pi deficiency, including one *PHT1;3*, two *PHT1;4*, two *PHT1;5*, and eight *PHT1;7* unigenes (**Figure 3B**), and these unigenes might play a dual role in Pi uptake and redistribution. For example, *OsPHT1;6* functions in Pi uptake and translocation throughout the plant (Ai et al., 2009). The plant cell vacuole is the main Pi storage pool, and VPEs are reported to export Pi from the vacuole to the cytosol; a previous study reported that VPEs mediate vacuolar Pi efflux in Pi-deprived *O. sativa* (Xu et al., 2019). Consistent with this, 10 *VPE1* and nine *VPE1* unigenes were found to be up-regulated in leaves and roots, respectively, and eight *VPE1* unigenes were co-up-regulated in both leaves and roots in Pi-deficient elephant grass (**Figure 3B**).

Taken together, the results suggest that up-regulation of *PAP*, *RNS*, *VPE*, and *PHT1* unigenes might enhance Pi acquisition and transport in elephant grass under Pi deficiency.

### 4.2 Recycling Pi *via* membrane lipids

Membrane lipid remodeling, defined as the replacement of membrane phospholipids (Pi-containing lipids) by Pi-free lipids, is a common response to Pi starvation in various plants (Sun et al., 2020). Membrane lipid remodeling involves two processes: phospholipid degradation and Pi-free lipid synthesis (Dissanayaka et al., 2018). A previous study reported that *phospholipase* genes were significantly up-regulated by Pi deficiency, especially *PLA*, *PLD*, *PAH*, *LysoPL*, and *GDPD* genes (Dissanayaka et al., 2021). Similarly, we also found many up-regulated *phospholipase* unigenes under Pi deficiency in elephant grass, including *PLA*, *PLD*, and *GDPD* unigenes (**Figure 5B**). Moreover, *phosphate ester enzymes* (*PAH* and *PECP* unigenes) were up-regulated, and their protein products can directly release Pi. Consistent with this, numerous Pi-containing lipids (phospholipids) decreased, including PC, PE, PG, and phosphatidylserine (PS; **Figure 4B**). Correlation analysis revealed that *phospholipase* unigenes were negatively correlated with Pi-containing lipids (**Figure 5C**). Therefore, Pi recycling might be enhanced in membrane lipid remodeling of elephant grass by phospholipid degradation (Dissanayaka et al., 2021).

The synthesis of Pi-free lipids was subsequently coupled to the degradation of phospholipids to maintain membrane integrity (Dissanayaka et al., 2018). Sulfo- and galactolipids are the main Pi-free lipids, which are used to replace phospholipids under Pi deficiency (Jeong et al., 2017). In elephant grass, Pi deficiency resulted in great increases in Pi-free lipids in both leaves and roots, including galactolipids (DGDG, MGDG, and DGMG), sulfolipids (SQDG) and glycerolipids (TG; **Figure 4B**). Consistently, unigenes that contributed to Pi-free lipids accumulation were up- or down-regulated by Pi deficiency; for example *MDG* (synthesis of MGDG) as well as *SQD1* and *SQD2* (synthesis of SQDG) unigenes were up-regulated, while *PLP* (degradation of MGDG) unigenes were down-regulated (**Figure 5B**). Correlation analysis demonstrated that *phospholipase* unigenes were positively associated with Pi-free lipids, and *PLP* unigenes were negatively correlated with Pi-free lipids (**Figure 5C**). Finally, the top 10 unigenes might play critical roles in regulating membrane lipid remodeling of elephant grass (**Figure 5D**).

Collectively, our results suggest that membrane lipid remodeling, including phospholipid degradation and replacing phospholipids with Pi-free lipids, might ensure efficient P utilization in elephant grass under Pi deprivation.

### 4.3 Conserving ATP and recycling Pi through bypasses in glycolytic pathway

In the cytosolic glycolysis pathway, some Pi- and adenylate-independent (such as PPi-dependent) reactions are employed to bypass reactions requiring Pi and/or adenylate under Pi deficiency conditions (Plaxton and Tran, 2011). Hydrolysis of PPi not only produces Pi, but also releases energy from high-energy phosphate ester bonds; hence PPi may help to conserve ATP and recycle Pi under Pi deprivation (Dissanayaka et al., 2021). In the present study, we identified five bypass reactions in elephant grass leaves under Pi deprivation, including up-regulated *sucrose synthase* and *UGPase* unigenes (PPi-dependent) were employed to bypass down-regulated *invertase*, *HK*, and *FK* unigenes (ATP-dependent); PPi-dependent unigenes (including *PPi-PFK* and *H^+^-PPase* unigenes) were recruited to bypass ATP-dependent bypassed unigenes (such as *ATP-PFK* and *H^+^-ATPase* unigenes) and up-regulated *PEPC* (involved in release of Pi), *MDH*, and *ME* unigenes were employed to replace down-regulated *PK* unigenes and non-significant *PPDK* unigenes (**Figure 6B**). Previous studies have reported that PPi can be used to release Pi by PPase hydrolysis (Dissanayaka et al., 2021). We also found that two *PPase* unigenes were up-regulated in Pi-deprived elephant grass leaves and roots (**Figure 6B**). These findings are in accordance with the results obtained in other Pi-deprived plants (Esfahani et al., 2020; Dissanayaka et al., 2021).

Overall, it is possible that PPi-dependent and Pi-independent bypass reactions are employed to replace adenylate- and/or Pi-dependent reactions in Pi-deprived leaves of elephant grass, thereby leading to conservation of ATP and recycling of Pi, and this strategy might achieve more efficient P utilization under Pi deficiency.

### 4.4 Increasing accumulation of phenylpropanoids and flavonoids

A large amount of phenylpropanoid and flavonoid metabolites was found to accumulate in response to Pi starvation in plants (Luo et al., 2020). These metabolites might influence root growth, enhance Pi availability, and perform antioxidant functions to adapt to low-Pi stress (Weston and Mathesius, 2013). For example, p-coumaric acid, ferulic acid, and naringenin were linked to the growth of roots (Weston and Mathesius, 2013). Genistein and quercetin were found to reduce and chelate iron, and thereby release Pi from iron phosphate (Tomasi et al., 2008; Cesco et al., 2010). Pi deficiency is expected to induce reactive oxygen species (ROS) accumulation and cause plants to suffer from oxidative stress, and flavonoids are reported to act as effective scavengers and inhibitors of ROS to protect plants (Agati et al., 2012). Consistent with earlier studies, several phenylpropanoids and flavonoids were observed to be remarkably elevated by Pi deficiency in elephant grass, such as phenylpropanoids (e.g. p-coumaric acid and ferulic acid) and flavonoids (e.g. naringenin and genistein 7-*O*-glucoside), in both Pi-deprived leaves and roots (**Figure 7C**, **Figure S5**). Consistent with these alterations, expression levels of unigenes (e.g., *PAL*, *C4H*, and *CHS* unigenes) involved in the biosynthesis of the aforementioned metabolites were largely up-regulated by Pi deficiency (**Figure 7A**). Furthermore, correlation analysis identified eight candidate unigenes positively associated with many phenylpropanoids and flavonoids, and these unigenes might play important roles in the accumulation of phenylpropanoids and flavonoids (**Figure 7D**). Importantly, MATE (subfamily II) members are known to transport flavonoids (Wang et al., 2016). In the present study, we detected eight *MATE* (subfamily II) unigenes that were up-regulated by Pi starvation in elephant grass leaves or roots (**Figure 7E**).

Overall, the phenylpropanoid and flavonoid metabolites markedly accumulated in Pi-deprived leaves and roots of elephant grass, and these metabolites might play an important role in root growth, rhizosphere Pi mobilization, and antioxidant activities under Pi starvation, but determining their specific roles requires further investigation.

## 5 Conclusions

**Figure 8:**
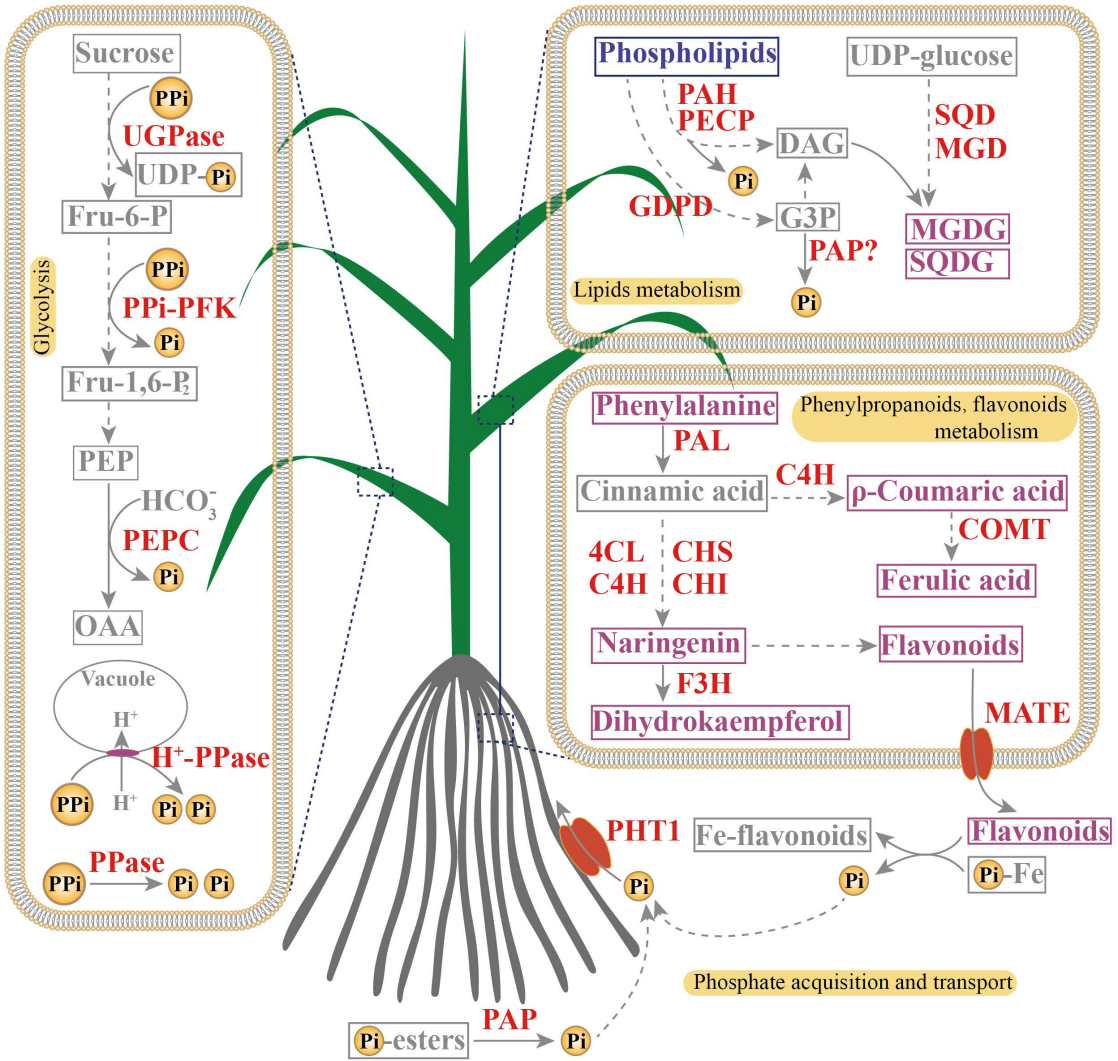
A proposed model of adaptive strategies to Pi deficiency in leaves and roots of elephant grass. Red indicate genes whose expression levels were up-regulated by Pi starvation. Purple and blue indicate metabolites whose accumulation increased (purple) or decreased (blue) in response to Pi deficiency.

In this study, integrated transcriptome and metabolome analyses were performed to investigate PSR unigenes and metabolites in leaves and roots of elephant grass. Numerous PSR unigenes involved in Pi acquisition and transport, Pi-containing lipid (phospholipid) degradation, Pi-free lipid biosynthesis, and phenylpropanoid and flavonoid metabolism were up-regulated in both Pi-deprived leaves and roots, and many Pi-independent and PPi-dependent unigenes were significantly increased in Pi-deficient leaves. Regarding metabolites, consistent with PSR unigenes, several phospholipids were decreased, and Pi-free lipids, phenylpropanoids, and flavonoids were increased in Pi-deficient leaves and roots. These findings suggest that facilitating Pi acquisition and transport, membrane lipid remodeling, Pi- and adenylate-independent bypassing reactions in glycolytic pathway, and phenylpropanoid and flavonoid accumulation may help to increase PAE and PUE in Pi-deficient elephant grass (**Figure 8**). Our findings will contribute to the development of more P-efficient elephant grass varieties.

## Data availability statement

The datasets presented in this study can be found in online repositories. The names of the repository/repositories and accession number(s) can be found in the article/**Supplementary Material**.

## Author contributions

JL and ZC: conceptualization and writing. RH: software and editing. YW: methodology. CL: software. CH: validation. PL and GL: review and editing. RD: writing – review and editing. All authors contributed to the article and approved the submitted version.
