# Supplementary material for: Integrated multi-omics reveals the molecular mechanisms underlying efficient phosphorus use under phosphate deficiency in elephant grass (Pennisetum purpureum)
Source: Front Plant Sci. 2022 Dec 23;13:1069191. doi: 10.3389/fpls.2022.1069191 (PMC9817030; doi:10.3389/fpls.2022.1069191)
Supplement: Supplementary file 1 [file DataSheet_1.docx]

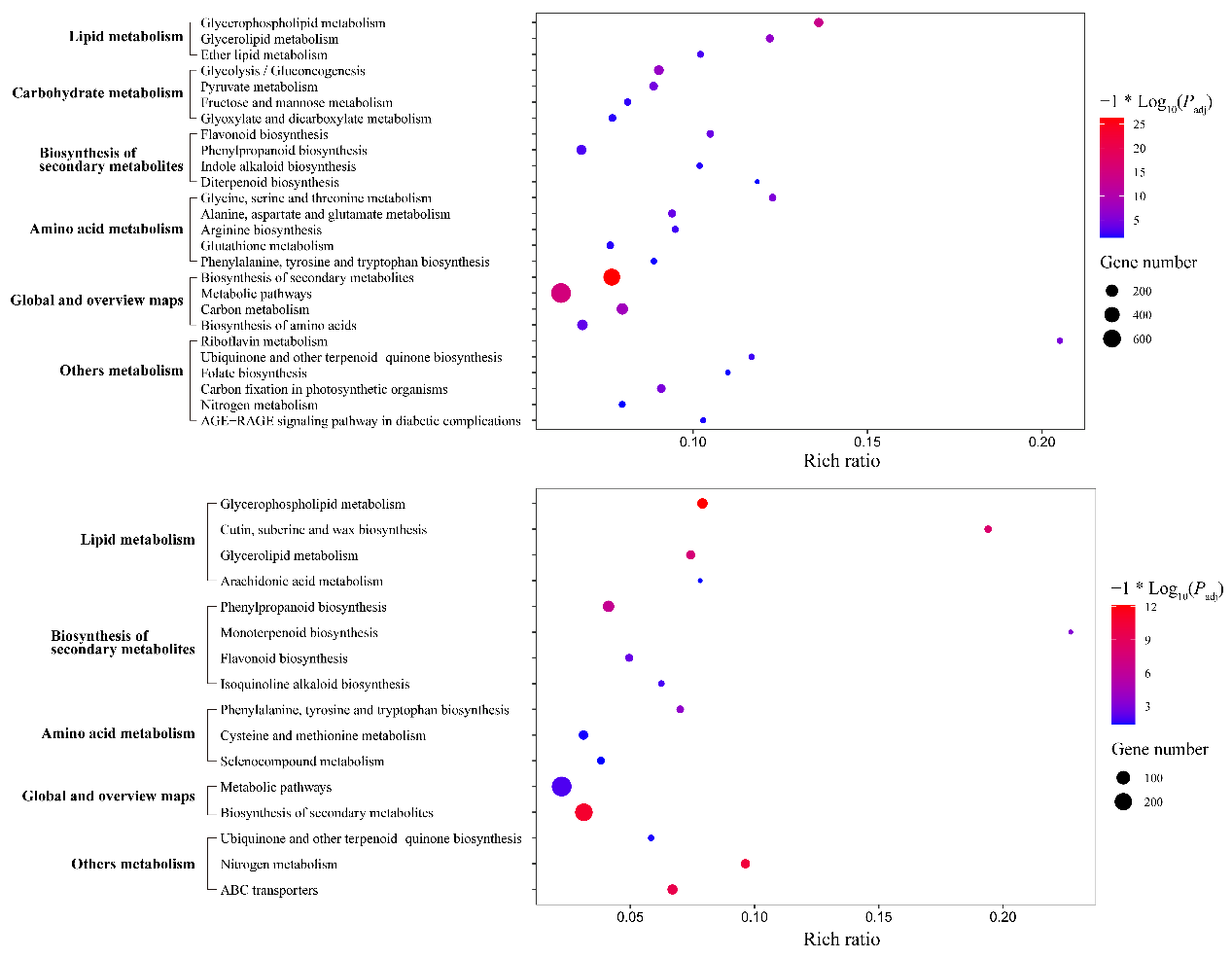


(A)

(B)

**Figure S1. All significant enriched KEGG pathways of differentially expressed unigenes (DEGs) in response to Pi starvation in leaves (A) and roots (B) of elephant grass.**


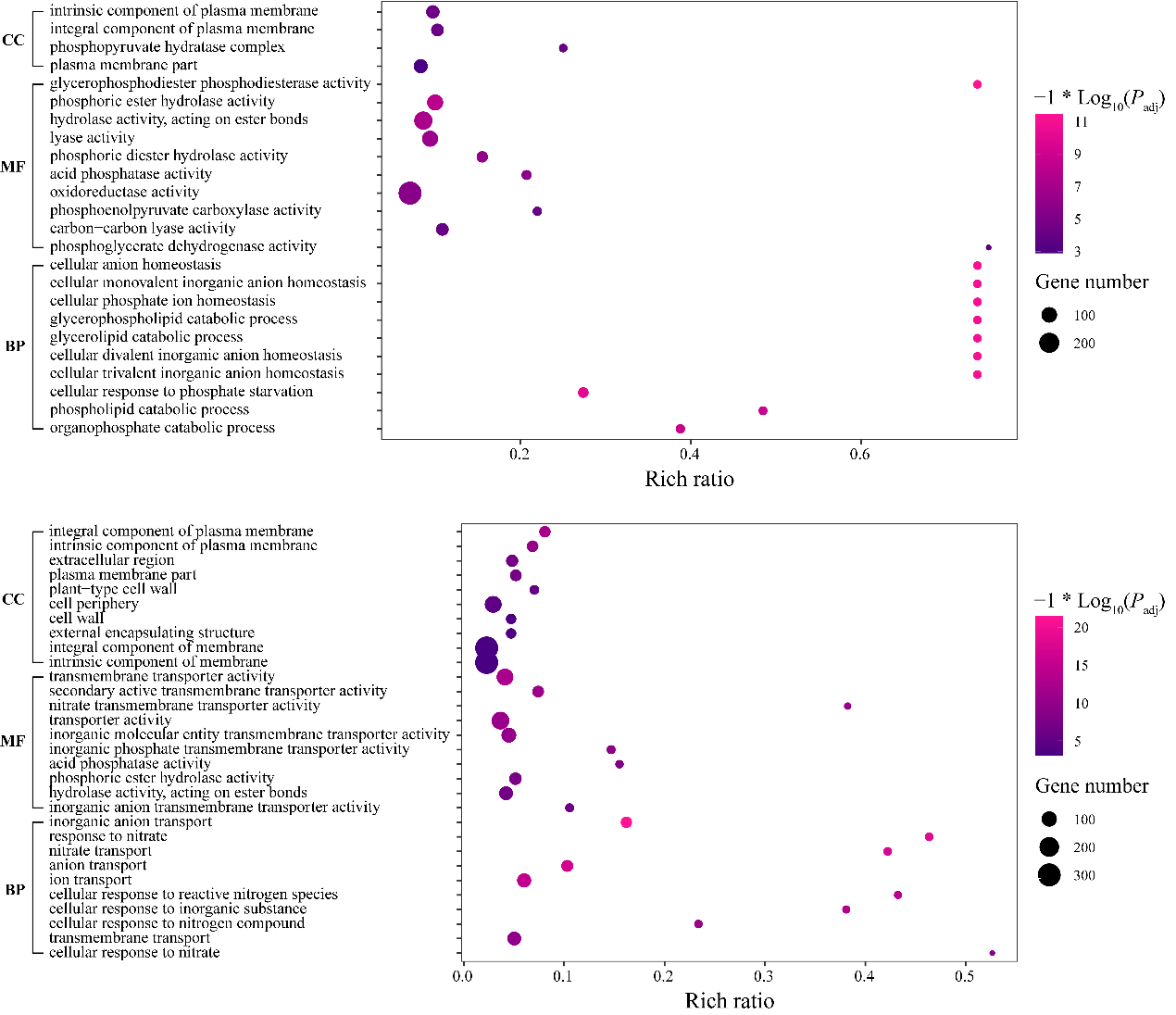


(A)

(B)

**Figure S2. Top ten of significant enriched terms associated with cellular component, molecular function and biological process of GO analysis.** These significant terms involved in DEGs upon Pi deficiency in leaves (A) and roots (B) of elephant grass.


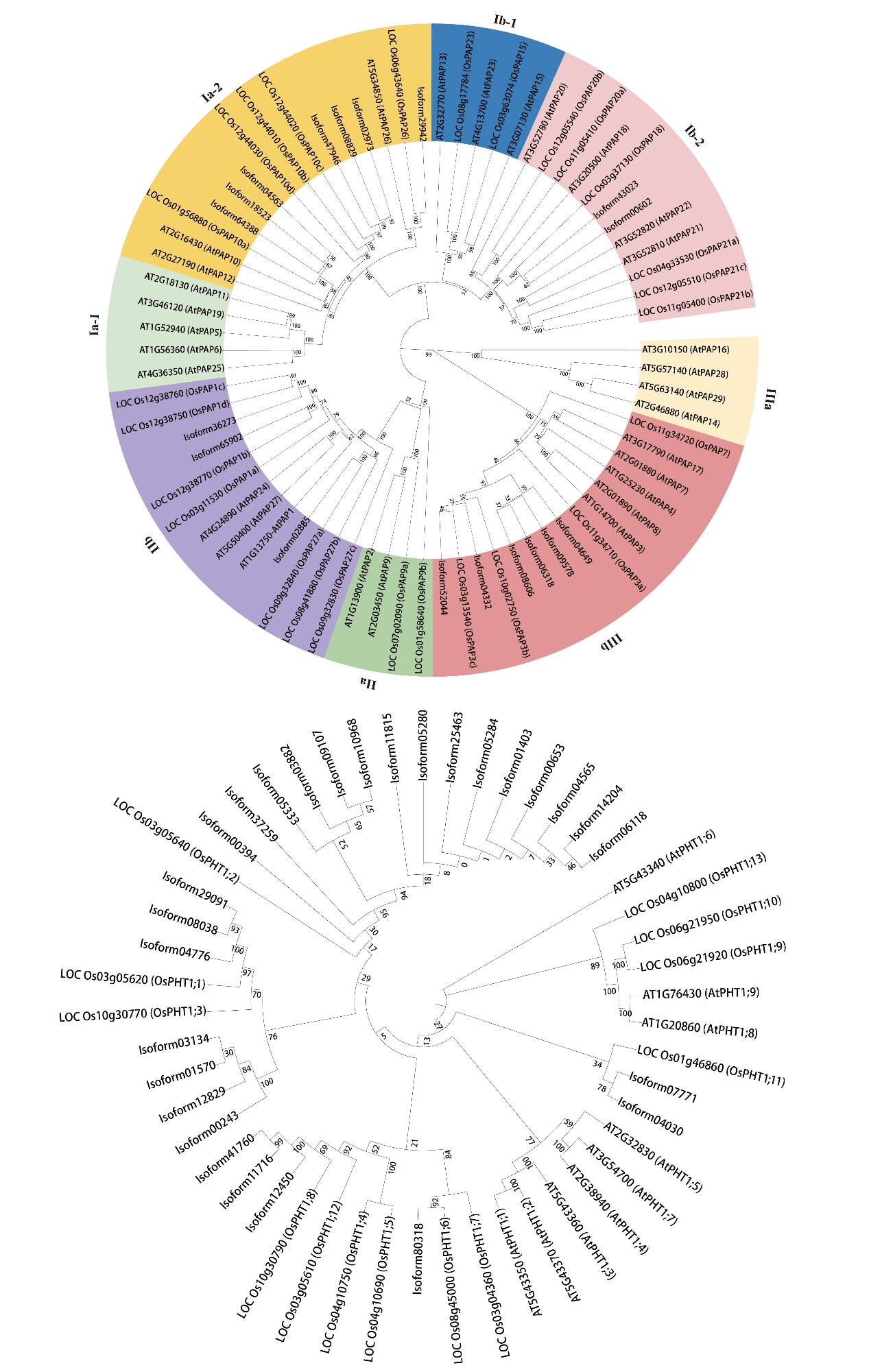


(A)

(B)

**Figure S3. Phylogenetic relationships of *PAP* and *PHT1* genes.** Protein phylogenetic trees were constructed among elephant grass, Arabidopsis thaliana and Oryza sativa PAP (A) and PHT1 (B) amino acid sequences.


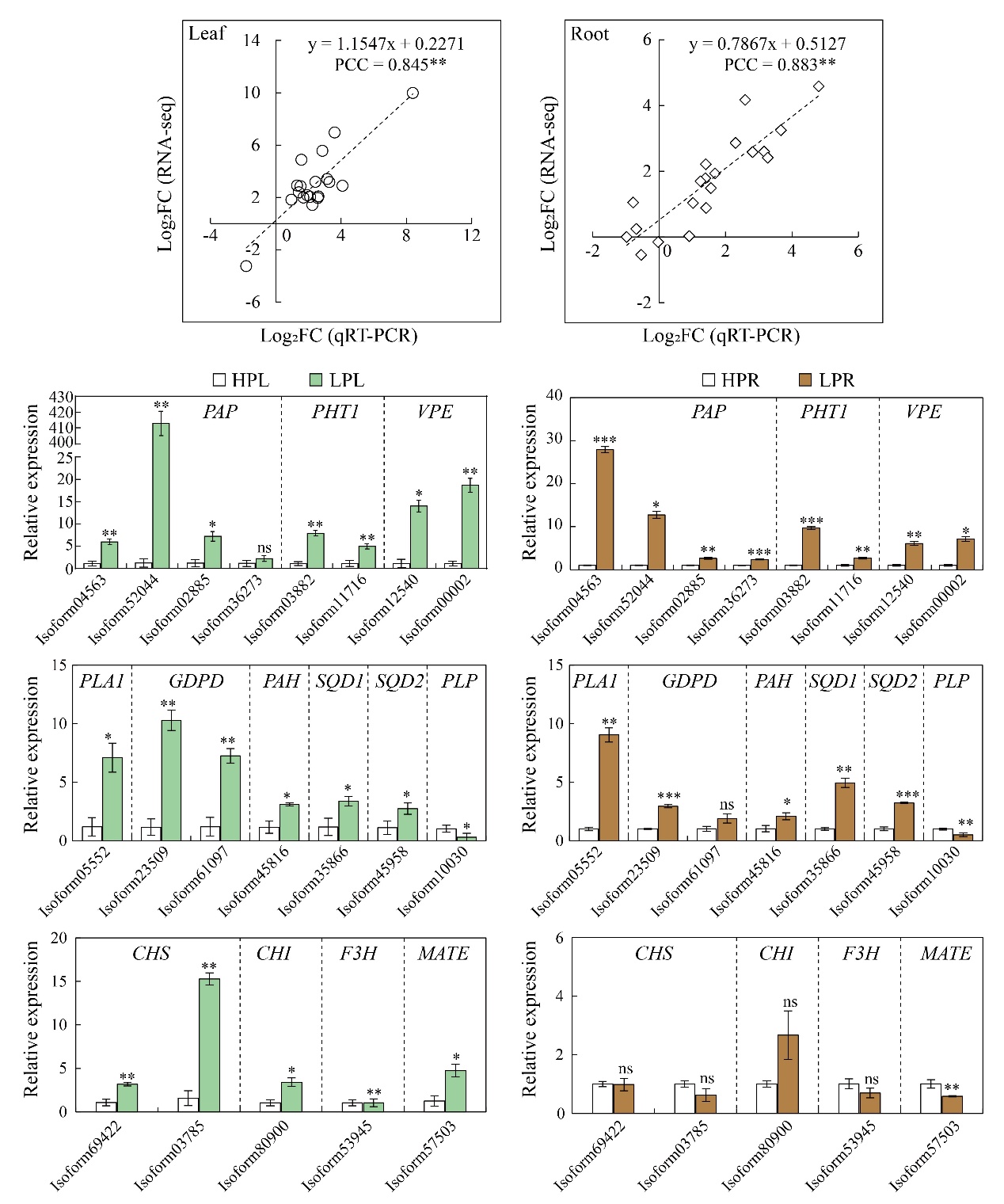


(A)

(B)

**Figure S4.** **Gene expression in quantitative real time PCR.** (A) Pearson’s correlation between comparative transcriptome (RNA-seq) data and quantitative real time PCR (qRT-PCR) data. (B) The relative expression levels in elephant grass leaves and roots of twenty unigenes by qRT-PCR.


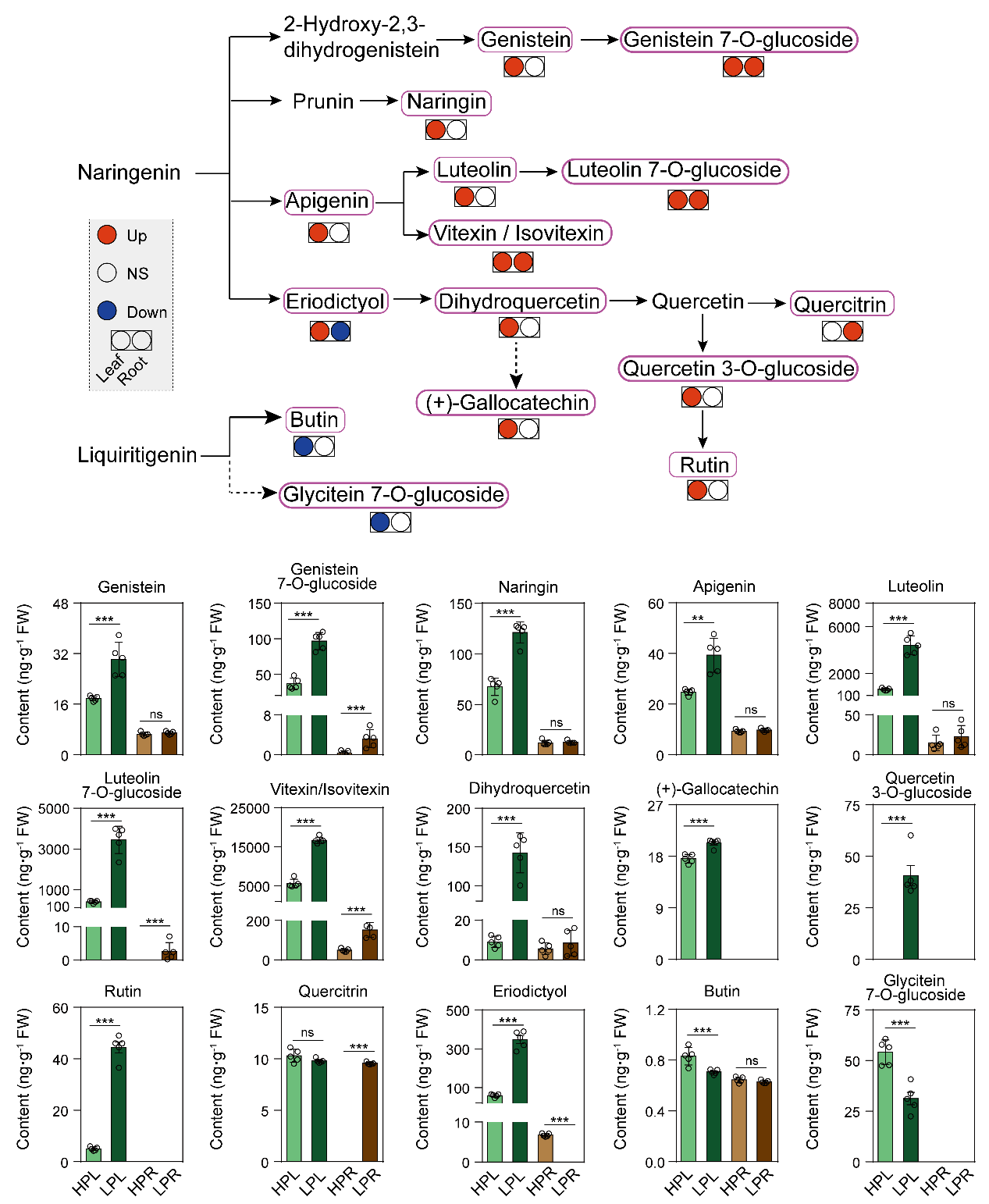


(A)

(B)

**Figure S5. Changes in the accumulation of flavonoid metabolites in elephant grass leaves and roots under low Pi stress.** (A) Statistics and network diagram of differentially accumulated metabolites. (B) Contents of flavonoids in leaves and roots. LPL: leaves grown under nutrient solution with 0 μmol·L-1 KH2PO4, HPL: leaves grown under nutrient solution with 600 μmol·L-1 KH2PO4, LPR: roots grown under nutrient solution with 0 μmol·L-1 KH2PO4, HPR: roots grown under nutrient solution with 600 μmol·L-1 KH2PO4.
